# Time-over-threshold readout to enhance the high flux capabilities of single-photon-counting detectors

**DOI:** 10.1107/S0909049511034480

**Published:** 2011-09-22

**Authors:** Anna Bergamaschi, Roberto Dinapoli, Dominic Greiffenberg, Beat Henrich, Ian Johnson, Aldo Mozzanica, Valeria Radicci, Bernd Schmitt, Xintian Shi, Laura Stoppani

**Affiliations:** a Paul Scherrer Institut, CH-5232 Villigen, Switzerland

**Keywords:** detectors, single-photon counting, time over threshold

## Abstract

The MYTHEN single-photon-counting (SPC) detector has been characterized using the time-over-threshold (ToT) readout method, *i.e.* measuring the time that the signal produced by the detected X-rays remains above the comparator threshold. In the following it is shown that the ToT readout preserves the sensitivity, dynamic range and capability of background suppression of the SPC mode, while enhancing the count-rate capability, which is the main limitation of state-of-the-art SPC systems.

## Abstract 2

The MYTHEN photon-counting ASIC operated in time-over-threshold mode shows an innovative approach towards the development of a detector operating with very high photon intensities while maintaining the single-photon sensitivity for synchrotron radiation experiments.

## 1. Introduction

Single-photon-counting (SPC) detectors detect individual photons as they arrive and add them to an internal counter for each channel. With the discriminator threshold set at a high enough level with respect to the electronic noise (Bergamaschi *et al.*, 2010 ▶), SPC detectors operate quasi-noiseless. Moreover, the detector threshold can be used to suppress the low-energy fluorescence radiation possibly emitted by the samples, therefore reducing the minimum detectable signal in applications where the useful information is dominated by background radiation flux (Ponchut & Zontone, 2003 ▶). In many synchrotron radiation experiments, despite the enormous input flux, a signal of a few photons may still constitute important information and the sensitivity and dynamic range provided by SPC detectors are extremely relevant (Lewis, 2003 ▶).

The main limitation of state-of-the-art counting systems appears at high photon fluxes. In fact, if a second photon arrives during the time required to register the previous one, it is lost and causes a loss of efficiency and of linearity. Although the data can be partially corrected, this effect sets a maximum limit on the count rate for the detector and consequently the data throughput is reduced.

Higher fluxes can only be measured with charge integrating (CI) detectors, which normally have disadvantages like limited dynamic range or a resolution that is not single-photon, sensitivity to sensor dark current and contribution to the background arising from the fluorescent radiation possibly emitted by the samples. The ideal detector would have the noise level, dynamic range and background suppression capability of a SPC detector, but the flux capability of a CI device.

In the following it will be shown that another method to extend the count-rate capability is to operate SPC devices using the time-over-threshold (ToT) acquisition mode while still preserving their outstanding dynamic range. ToT is a well known method in high-energy-particle detectors, mainly used for particle identification (Akesson *et al.*, 2001 ▶). It allows the energy deposited by a particle in the detector element to be estimated by measuring the time during which the signal generated by the detected particle remains above a comparator threshold. The ToT method directly converts the signal pulse height into a digital value in the early stage of the front-end electronics in parallel for all the channels of the detector, which greatly simplifies the system compared with analog detectors with serial readout through one or several ADCs. ToT has also been implemented for hybrid X-ray detectors (TimePix) by Llopart *et al.* (2007 ▶) and tested for a wide range of applications (Jakubek, 2010 ▶).

In the following, issues concerning the response of the MYTHEN SPC detector operated in ToT mode are discussed in the case of synchrotron radiation experiments. Since the MYTHEN analogue signal shape is not optimized for ToT applications, the goal of the measurements is not to obtain spectroscopic information for each single detected photon, like in, for example, fluorescence imaging as described by Zemlicka *et al.* (2009 ▶), but instead to enhance the photon-counting capability in diffraction and imaging experiments at high count rates.

## 2. Material and methods

### 2.1. The detector

**Figure 1:**
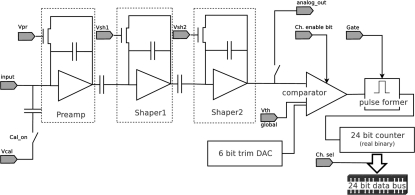
Sketch of the architecture of a channel of the MYTHEN front-end electronics.

The MYTHEN (microstrip system for time-resolved experiments) detector has been developed for synchrotron radiation powder diffraction experiments (Bergamaschi *et al.*, 2010 ▶). The front-end integrated circuit consists of 128 channels operating in parallel in single-photon-counting mode (Mozzanica *et al.*, 2009 ▶). The architecture of one channel is sketched in Fig. 1 ▶. It mainly consists of a charge-sensitive preamplifier AC-coupled to two shaping gain stages followed by a comparator, a pulse generator and a counter.

The analogue chain can be tuned, and three different settings have been defined for MYTHEN in order to cover a large range of applications for what concerns the minimum detectable X-ray energy, which depends on the electronic noise, and the maximum count rate, which depends on the shaping time, as described in detail by Bergamaschi *et al.* (2010 ▶):

(i) *High gain* settings are optimized for energies down to 7 keV (5 keV with some efficiency loss) and count rates only up to about 100 kHz per channel;

(ii) *Fast* settings are optimized for count rates up to 900 kHz per channel but energies only down to 10 keV;

(iii) *Standard* settings match most synchrotron radiation applications with regards to both the energy range (down to 8 keV) and the count rate (up to 500 kHz per channel).

The comparator threshold can be trimmed on a channel-by-channel basis by means of an internal 6-bit digital-to-analog converter (DAC) which adds to the global externally adjustable threshold to reach a threshold dispersion down to about 100 eV. Each comparator output is fed into a 24-bit gateable counter. In addition, the chip contains the digital logic for configuring the internal DACs, resetting and reading out the counters serially over four parallel data output lines. The counters are read out using a 16 MHz clock and the dynamic range of the counter can be reduced in order to decrease the readout time. Frame rates ranging between 4 kHz (24-bit) and 14 kHz (1-bit) are achievable.

The pulse former following the comparator generates a pulse only when the comparator output is in coincidence with an external digital signal (gate) which acts like an electronic shutter. By applying an oscillating gate, a pulse is generated for every cycle while the signal is above threshold and the detector can be operated in ToT mode. The gated clock is generated in the firmware by dividing a 200 MHz clock and its frequency is adjustable by software but limited to a maximum of 50 MHz owing to the finite width of the count pulse generated after the comparator. Several clock frequencies in the range 10–50 MHz have been tested, with similar outcomes. In the following, only the results obtained for 50 MHz will be shown.

The detector used for the measurements is a standard MYTHEN module and consists of ten ASICs (application-specific integrated circuits), *i.e.* 1280 independent channels wire-bonded to a 50 µm-pitch sensor with 8 mm-long strips. The detector is controlled by a socket interface *via* TCP/IP and custom firmware, and software have been implemented in order to perform the acquisition and readout of the detector. Switching between counting and ToT mode is performed by a single software command.

### 2.2. The beamline

The data presented in this paper have been acquired at the medical imaging beamline SYRMEP at the Elettra synchrotron light source in Trieste, Italy (Abrami *et al.*, 2005 ▶). The beamline is particularly suitable for detector characterization owing to its wide laminar beam with a maximum area of about 160 mm × 5 mm at the experimental station, which allows the whole detector module to be irradiated at once. The beam is monochromatic in the energy range 8–35 keV with an energy resolution of about 0.1% which is negligible compared with that of the detector. The typical flux measured at the sample position at 17 keV is about 1.6 × 10^8^ γ mm^−2^ s^−1^, with a stored electron beam of 300 mA at 2 GeV. The filling pattern of the accelerator is given by 432 electron bunches spaced 2 ns and for the purpose of this characterization can be well approximated by a continuous flux of Poisson-distributed X-rays. For the measurement of the rate response of the detector the beam can be attenuated with a system of aluminium filters and its intensity can be monitored by using a calibrated ionization chamber. Micrometric translation and rotation stages allow positioning and scanning of the samples and detectors with respect to the stationary beam for the spatial resolution measurements.

## 3. Experimental results

A module of the MYTHEN detector operated in ToT mode has been characterized in detail and compared with its SPC behavior in terms of noise, count rate and spatial resolution. The spectroscopic behavior of the detector will not be addressed in the following since its energy resolution is not sufficient for spectroscopic experiments and the system has the disadvantage of a long readout time compared with CI detectors such as, for example, the one described by Mozzanica *et al.* (2010 ▶). Since the ToT response is very sensitive to variations in the shaping time of the analogue signal and MYTHEN was not optimized to minimize these mismatches, the performance of single channels has been analyzed individually. In the following the results relative to one channel of the detector showing an average performance (channel number 645) will be discussed, but they can be generalized to all channels by using different parameters.

The analogue signal *S* as a function of time *t* can be approximated by a semi-exponential function with decay time τ and amplitude *E* proportional to the energy deposited by the X-rays and collected by the detector element,
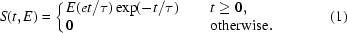
The ToT response *w* of the detector is given by the difference between the two solutions of the equation 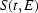 = 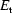, where 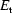 is the threshold of the detector,
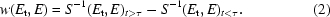
In the following it will be assumed that the energy spectrum measured by hybrid detectors for a monochromatic X-ray beam of energy 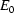 can be written as described by Bergamaschi *et al.* (2008 ▶),
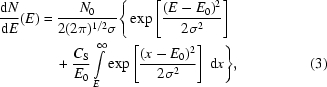
where 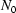 is the total number of photons, σ is the equivalent noise charge (ENC) of the detector, *i.e.* the electronic noise in terms of the input signal needed to obtain the same output at the end of the analogue chain (Radeka, 1988 ▶), and 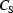 is a coefficient which represents the charge sharing, *i.e.* the fraction of photons which produce a charge cloud which is shared between neighboring strips, mainly owing to the diffusion of the charge while it drifts towards the electrodes.

### 3.1. Threshold scans

In order to compare with SPC data, threshold scans (*i.e.* measurements monitoring the response of the detector while changing its threshold) have been acquired in both SPC and ToT mode.

**Figure 2:**
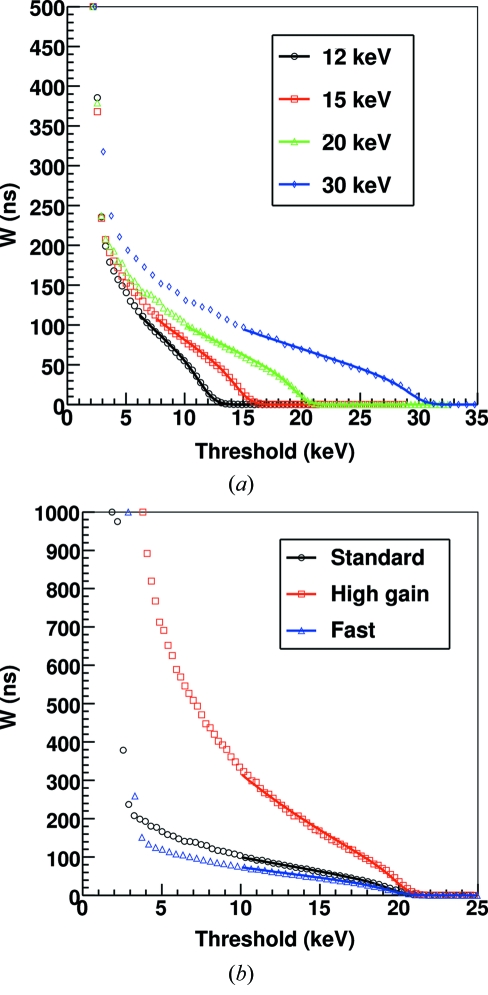
Threshold scans of one channel of the detector acquired in ToT mode for (*a*) different energies, standard settings and (*b*) different settings, 20 keV. The fits of the experimental points with the model of equation (5) are shown by the solid lines and the resulting parameters are listed in Table 1 ▶.

In SPC detectors the threshold scan curve 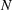 is given by the integral of (3) above the threshold 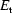,
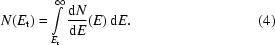
In the case of a ToT detector the threshold scan curve 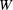 is given by the product of the energy spectrum measured by the detector [equation (3)] with the conversion function [equation (2)] integrated for all the energies above the threshold 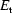,
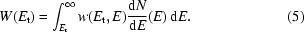
In general, for each threshold it will be possible to define a conversion factor λ,
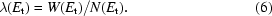
Initially, threshold scans acquired in SPC mode have been used in order to calibrate the threshold-to-energy conversion of each channel of the detector as described in detail by Bergamaschi *et al.* (2010 ▶). By fitting the curves acquired in ToT mode, it is possible to estimate τ of the analogue signal and ENC of the detector. The results for the different settings are listed in Table 1 ▶. Some example of fits for different energies and settings for channel 645 are shown in Fig. 2 ▶. The ToT threshold scans fit the curves of equation (5) with the ENC estimated by SPC kept as a fixed parameter, showing that the electronic noise is not strongly affected by the presence of the clocked gate and proving that MYTHEN operating in ToT mode can reach the same low energies and suppress the fluorescent background as in SPC mode.

### 3.2. Statistical significance of counts

**Figure 3:**
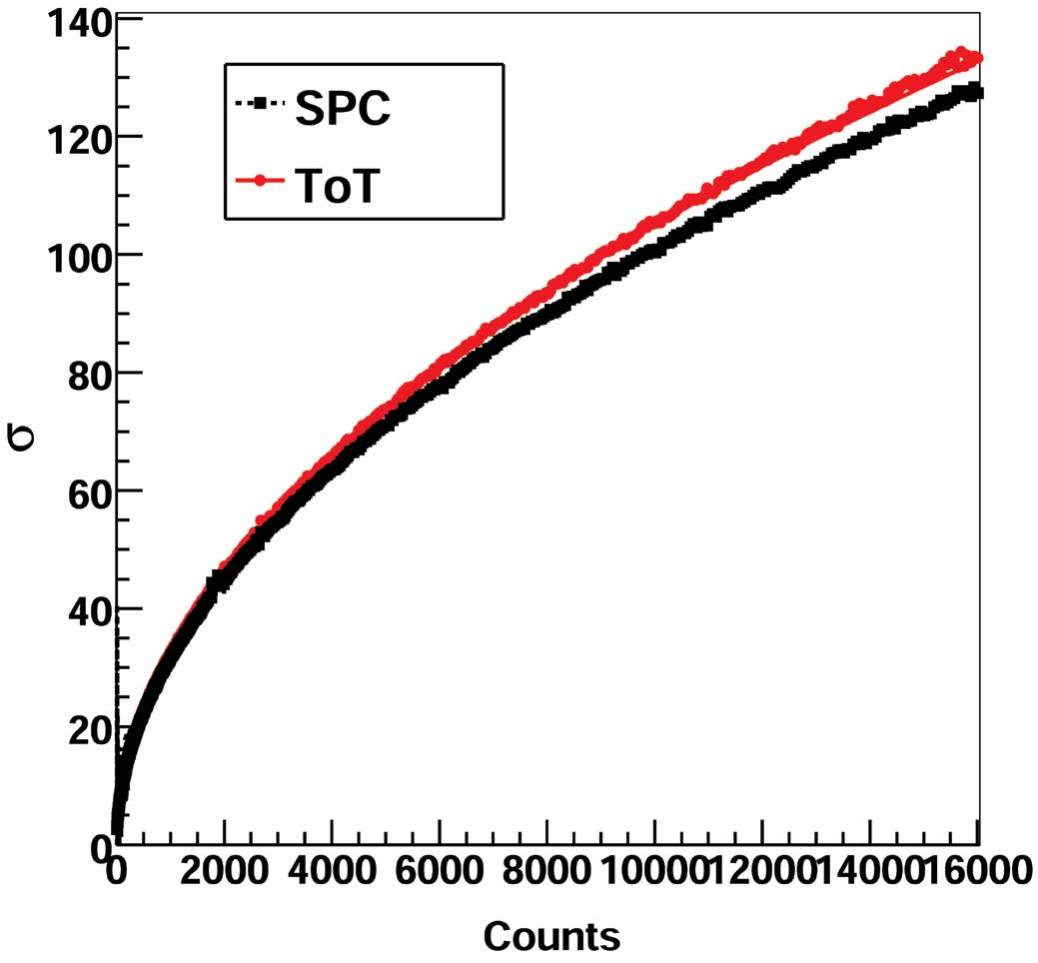
Average standard deviation on the number of counts per channel in SPC (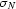) and ToT (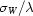) mode measured using Ag fluorescence radiation (22 keV) with standard settings and threshold set at half of the X-ray energy as a function of the number of counts 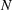. The number of counts has been changed by changing the acquisition time.

In a counting detector the error on the number of counts is purely given by the Poisson statistics,
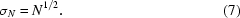
In ToT mode the error on 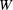 is given by the combination of the Poisson statistics and of the error 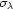 on the conversion factor λ [equation (6)] which is due to the distribution of the collected charge owing to charge sharing (Jakubek, 2009 ▶), to the ENC of the detector (Manfredi *et al.*, 2000 ▶) and to the finite frequency of the clocked gate,
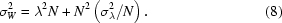
Therefore the fluctuations on the number of counts in ToT mode is increased compared with SPC by a factor which is smaller for higher clock frequencies and smaller ENC,
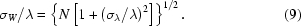
Fig. 3 ▶ shows the fluctuations on the number of photons measured in SPC (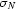) and ToT (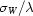) mode as a function of the number of counts 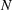. The data have been acquired with standard settings by measuring the radiation produced by a fluorescent Ag sample 500–10000 times while changing the acquisition time in the range 5 ms to 10 s keeping the threshold fixed at half of the X-ray energy. Similar results have been obtained with different settings and thresholds. The data have been fitted with a function σ = 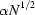 and the estimated parameters are 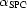 = 1.0107 ± 0.0001 and 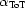 = 1.049 ± 0.005. The increase on the fluctuations at higher statistics (and therefore acquisition times) can be explained by taking into account the stability of the source (X-ray tube) and detector, since the measurement lasted for several days. From equation (9) one can estimate 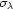 ≃ 23 ns for 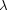 = 89 ± 3 ns for standard settings. Although 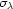 is relatively large, the uncertainty added to the number of counts is small (∼5%) compared with the intrinsic Poisson fluctuations and therefore the counting statistics of the detector are not strongly affected.

### 3.3. Rate response

Normally a detection system is characterized by a dead-time, defined as the time after each detected event during which the system is not able to record another event if it happens (Leo, 1994 ▶). In a paralizable detector an event happening during the dead-time of the previous event is missed and restarts the dead-time, so that with increasing rate the detector reaches a saturation point where it is incapable of recording any event at all. However, in a non-paralizable detector, an event happening during the dead-time since the previous event is lost but does not extend the dead-time, so that with an increasing event rate the detector reaches a saturation rate equal to the inverse of the dead-time but is always sensitive to radiation.

**Figure 4:**
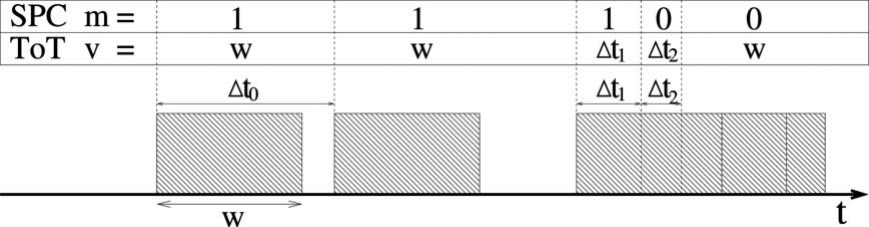
Diagram explaining the response of a detector for a time interval between photons 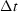 longer (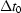) or shorter (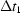, 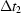) than the time that the signal remains above threshold 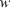. The response of a SPC detector is indicated by 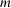 [equation (10)] and that of a ToT detector by 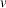 [equation (12)].

In the case of SPC systems a deviation from the linearity of the number of counts occurs at high photon fluxes because of the pile up of the analog signal generated by the X-rays absorbed in a very short time in the same strip. Fig. 4 ▶ compares the response of a SPC and a ToT detector for different arrival times of the X-rays. This simplified model uses a fixed time 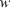 during which the signal remains above threshold, without taking into consideration that the charge produced by the second photon piles up with the charge still present on the preamp feedback capacitor, and therefore the time above threshold owing to the second photon is longer than 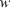, depending on the shaping of the analog signal and on the threshold level.

A SPC detector behaves like a paralizable system with dead-time given by the time 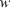 that the analogue signal remains above the threshold. The response 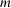 to the latest photons for a SPC detector, where 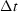 is the time interval to the previous event, is
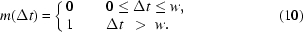
By averaging over the Poisson-like statistics of an X-ray beam of flux 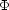 = *n*/*T* (where 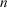 is the number of photons absorbed in the detector element in the time interval 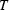), one obtains the counting efficiency 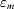 of a paralizable detector,
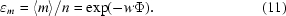

               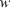 places a maximum limit for the intensity of the beam above which it is impossible to correct for the loss of efficiency at 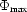 = 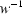.

**Figure 5:**
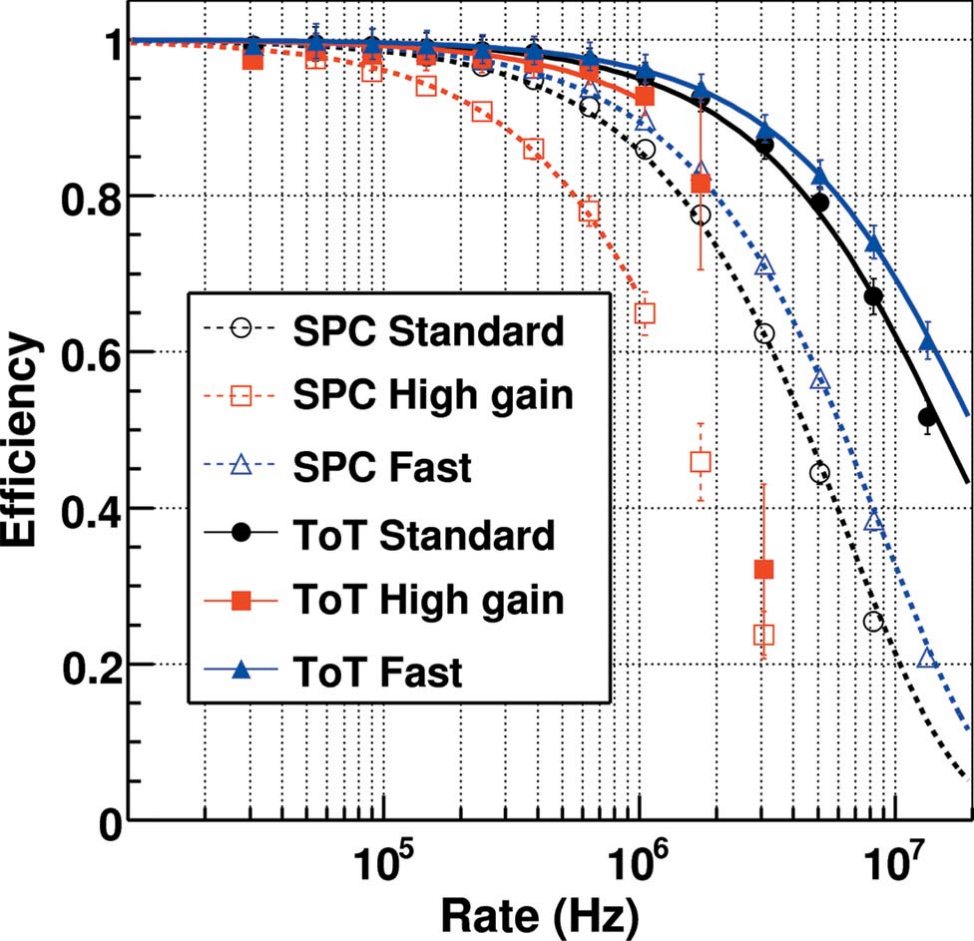
Average efficiency of the MYTHEN detector operated in SPC and ToT mode as a function of the rate for the different settings at 15 keV with the threshold set at 7.5 keV. The SPC data are fitted with equation (11) while the ToT data are fitted with equation (13). For *high gain* settings the data are fitted only up to 1 MHz since, for higher rates, saturation effects appear and the detector behavior deviates from the ideal paralizable detector. See also Table 2 ▶.

On the other hand, in ToT mode when two photons pile up, the signal remains above the threshold level for a longer period and the loss of efficiency is therefore reduced compared with the SPC mode. As sketched in Fig. 4 ▶, the response 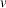 to a photon in the case where a second one comes after the interval 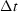 is 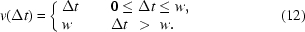
By averaging over the Poisson-like time of arrival of the X-rays one obtains the ToT efficiency 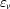,
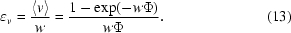
As expected, 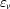 is always larger than 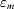 with the same 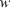. It is important to point out that the efficiency of a detector operated in ToT mode does not have any limit on the maximum count rate, and at 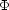 = 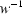 the efficiency in ToT mode is still higher than 60%. Although the efficiency of the detector operated in ToT mode is reduced at high rates, the system does not have a fixed dead-time during which the detector is completely insensitive to radiation, and therefore its high rate response is improved even compared with the efficiency 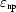 of a non-paralizable detector with dead-time 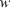 (Leo, 1994 ▶),
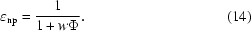
Fig. 5 ▶ shows the measured efficiency of the detector in SPC and ToT mode as a function of the X-ray rate for the different settings at 15 keV with the threshold set at 7.5 keV. The number of counts 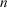 has been estimated by using the calibrated ionization chamber available at the SYRMEP beamline. Table 2 ▶ highlights the count rates at which the efficiency loss exceeds 1% and 10% and it is evident that the fluxes for the same efficiency are higher in ToT with respect to SPC mode by a factor of 2.5–5 depending on the settings.

The estimated 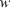 for different energies and settings are listed in Table 3 ▶. Since the residual charge on the feedback capacitor is neglected, the efficiency loss is underestimated in the case of SPC [equation (11)], but overestimated in the case of ToT [equation (13)]. For this reason the measured values for 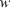 are always lower for the ToT with respect to the SPC mode. The increasing behavior of the counting efficiency (negative 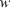) at higher energies (20 keV) in the case of *high gain* settings can be explained by the saturation of the preamp at high rates. However, an optimization of the shaping of the analogue signal for ToT operation can bring a more stable behavior over the whole energy range.

**Figure 6:**
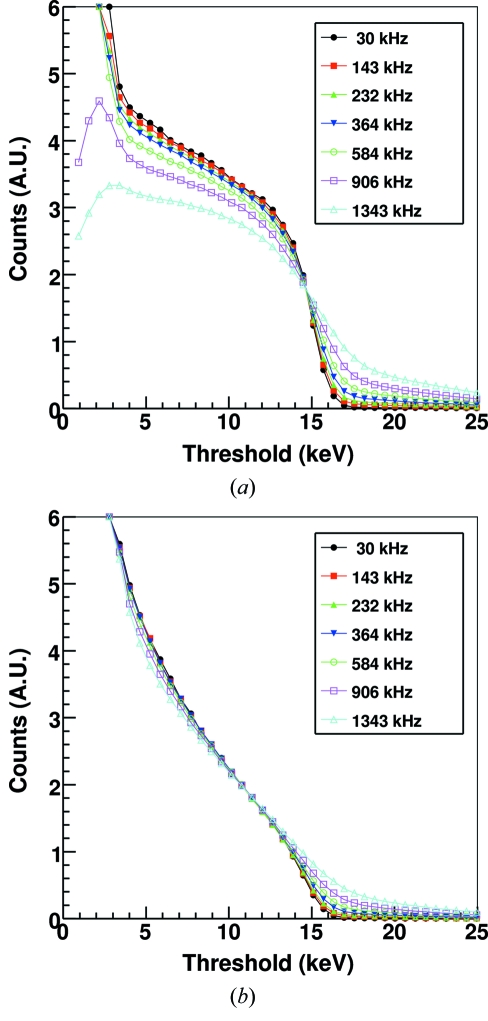
Threshold scans acquired at 15 keV and different X-ray fluxes using standard settings in (*a*) SPC and (*b*) ToT mode for channel 645. The number of counts has been normalized to the ionization chamber readout.

Fig. 6 ▶ shows the counting response as a function of the counter threshold with different X-ray intensities in SPC and ToT mode at 15 keV with standard settings for channel 645. The number of counts has been normalized to the ionization chamber readout. It is evident that up to about 500 kHz the rate corrections required at all thresholds in ToT are negligible, which is a big advantage compared with SPC where the choice of the required correction coefficients should be performed as a function of the X-ray energy, threshold level and fill pattern of the electron ring.

Moreover, the increase of the efficiency obtained with *high gain* settings in ToT mode opens the possibility to work at high rates (100 kHz to 1 MHz) also at low X-rays energies with small rate corrections, which was not possible with the SPC operation of the detector.

### 3.4. Spatial resolution

The spatial resolution of the detector operated in SPC and ToT mode has been evaluated by measuring the edge spread function (ESF), which in a one-dimensional detector represents the integral of the point spread function (PSF). This has been obtained by scanning an edge in front of the detector and averaging the response of the channels as a function of the relative position of the edge from the center of a strip as explained in detail by Samei *et al.* (1998 ▶).

**Figure 7:**
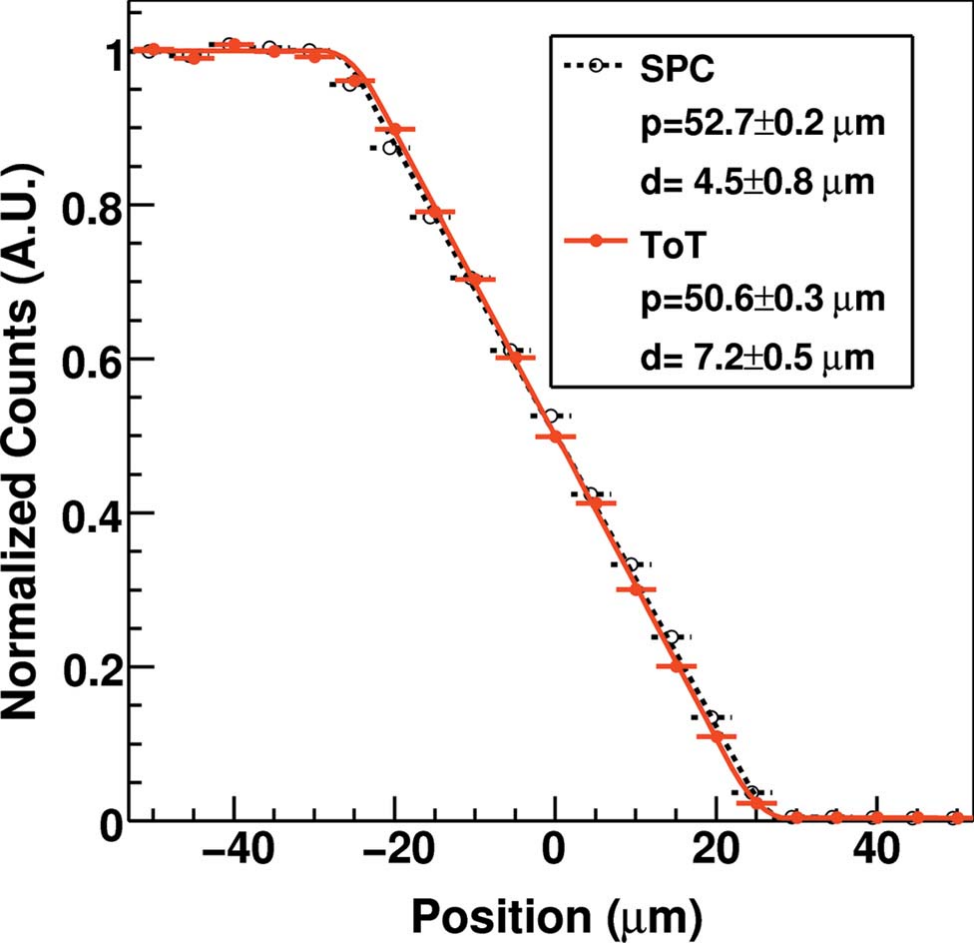
Edge spread function of channel 645 acquired at 15 keV with the threshold set at half of the X-ray energy and *standard* settings in SPC and ToT mode. The estimated values for the ESF obtained by fitting the experimental points in SPC and ToT mode with a trapezoidal function are reported in the legend. 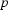 is the FWHM of the trapezoid while 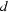 is the difference between its bases.

Fig. 7 ▶ shows the ESF measured at 15 keV with the threshold set at half of the X-ray energy and *standard* settings while scanning an edge in 5 µm steps in front of channel 645 operated in SPC and ToT mode. The experimental points have been fitted with the ESF curve corresponding to a trapezoidal PSF as described by Bergamaschi *et al.* (2008 ▶). The estimated values for the ESF are reported in the legend, where 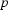 is the FWHM of the trapezoidal PSF and should correspond to the strip pitch, while 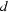 is the difference between the bases of the trapezoid and represents the charge-sharing region.

It is evident that the spatial resolution is not strongly affected by the operation mode. The small difference in the estimated 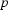 and the larger value of 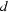 obtained for the ToT readout are due to the fact that the PSF in ToT readout mode is not easily described by the trapezoidal model. In fact, while SPC detectors assign the same weight to each signal above the threshold, the response of the detector in ToT mode is sensitive to the amount of charge collected for each photon. In the region between the strips, when charge-sharing occurs, the weight assigned to the photon is therefore sensitive to the absorption position. Data acquired with sensors with smaller strip pitches are under analysis in order to study the feasibility of interpolating between neighboring strips (Bergamaschi *et al.*, 2011 ▶). Similar results have been obtained for different energies, settings, gated clock frequencies and threshold values.

## 4. Conclusions

The performance of the MYTHEN detector operated in SPC and ToT mode have been characterized and compared. It has been shown that the ToT operation does not significantly affect the noise of the detector and therefore allows operation down to 5 keV energy as in SPC mode. Moreover, compared with CI detectors, ToT operation still allows the suppression of the fluorescence background possibly produced by the sample. The spatial resolution is also comparable in both operation modes.

Although the counting statistics are slightly deteriorated in ToT mode, single-photon resolution is still possible and the fluctuations on the number of counts is always mainly due to the intrinsic Poisson fluctuations.

The improved performances of the detector at high count rates strongly motivate the use of ToT mode in synchrotron radiation experiments. The rate capability of MYTHEN is extended by at least a factor of two when using the ToT acquisition mode and this can further be optimized by using a different shaping of the analogue signal. MYTHEN can be operated with *high gain* settings (low noise) with improved performances at high rates compared with the *standard* or *fast* settings of traditional SPC operation and, therefore, experiments can be performed at low energies also with high radiation fluxes, without the need to attenuate the beam as would be required in SPC mode.

Applications which can also benefit from the improved count rate are in particular high-rate applications where rate corrections are not properly usable in SPC mode like, for example, single-crystal fine ϕ-sliced diffraction experiments (Pflugrath, 1999 ▶) or in the case of asymmetric fill patterns of the synchrotron ring (Bateman, 2000 ▶; Walko *et al.*, 2008 ▶).

The main disadvantage of ToT is the need to calibrate each channel of the detector individually. However, this step can be easily performed by acquiring a flat field in operating conditions both in ToT and SPC mode at the beginning of the experiment and motivates the development of detectors with two independent counters working in parallel, respectively, in ToT and SPC mode. In this case a simple flat-field acquisition at low rates allows the calibration of all channels simultaneously.

## Figures and Tables

**Table 1:** Estimated ENC and τ for one channel of the detector from the fits of the threshold scan curves with the model of equation (5) The ENC values are derived from SPC data and are then kept as a fixed parameter in the fit with the ToT threshold scans.

Settings	ENC (e^−^)	τ (ns)
Standard	230 ± 10	85.2 ± 0.4
High gain	210 ± 10	214.0 ± 0.1
Fast	260 ± 10	54.9 ± 0.1

**Table 2:** Fluxes at which the efficiency of the MYTHEN detector are 99% or 90% for the different settings and readout modes as calculated from the fitted functions

	Φ (kHz) at 15 keV			
	∊ = 99%		∊ = 90%	
Settings	SPC	ToT	SPC	ToT
Standard	65 ± 1	194 ± 10	684 ± 13	2070 ± 110
High gain	22 ± 1	126 ± 40	260 ± 13	1300 ± 500
Fast	90 ± 1	260 ± 13	940 ± 16	2760 ± 140

**Table 3:** Time during which the signal remains above threshold 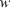 for different settings and energies with the threshold set at half of the photon energy The SPC data are fitted with equation (11) while the ToT data are fitted with equation (13).

	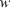 (ns)					
	12 keV		15 keV		20 keV	
Settings	SPC	ToT	SPC	ToT	SPC	ToT
Standard	161 ± 4	142 ± 9	154 ± 3	104 ± 5	128 ± 6	31 ± 14
High gain	418 ± 32	329 ± 100	401 ± 22	160 ± 53	327 ± 21	−229 ± 89
Fast	118 ± 3	106 ± 8	112 ± 2	78 ± 4	85 ± 5	23 ± 12
